# Management of poor-prognosis testicular germ cell tumors

**DOI:** 10.4103/0970-1591.61228

**Published:** 2010

**Authors:** Kiranpreet Khurana, Timothy D. Gilligan, Andrew J. Stephenson

**Affiliations:** Glickman Urological and Kidney Institute, Cleveland Clinic, Cleveland, OH; Department of Solid Tumor Oncology, Taussig Cancer Center, Cleveland Clinic, Cleveland, OH, USA

**Keywords:** Testicular neoplasms, neoplasms, germ cell and embryonal, antineoplastic combined chemotherapy protocols, retroperitoneum, lymph node excision, prognosis, risk factors, salvage therapy

## Abstract

Currently, the outcome of patients with intermediate-and poor-risk germ cell tumors at diagnosis is optimized by the use of risk-appropriate chemotherapy and post-chemotherapy surgical resection of residual masses. Currently, there is no role for high-dose chemotherapy in the first-line setting. Patients who progress on first-line chemotherapy or who relapse after an initial complete response also have a poor prognosis. In the setting of early relapse, the standard approach at most centers is conventional-dose, ifosfamide-based regimens and post-chemotherapy resection of residual masses. The treatment of patients with late relapse is complete surgical resection whenever feasible. Salvage chemotherapy for late relapse may be used prior to surgery in patients where a complete resection is not feasible. A complete surgical resection of all residual sites of disease after chemotherapy is critical for the prevention of relapse and the long-term survival of patients with advanced germ cell tumors.

## INTRODUCTION

In the United States, testicular germ cell tumor (GCT) is the most common malignancy among men aged 20-40 years. With the development of cisplatin-based chemotherapy and the integration of surgery, GCTs have become a model of a curable neoplasm.[1] In the pre-cisplatin era, cure rate for patients with advanced GCT was 5-10%. Currently, the long-term survival for men with advanced GCT is 80-90%. While the outcome for the vast majority of GCT patients is favorable, an estimated 380 men will die from testis cancer in 2009 in the United States.[2] Any mortality from GCT is a tragic occurrence given the relative young age of this patient population. The average years of potential life lost per GCT death is 33 years, which is among the highest of all adult cancers.[3]

Mortality from GCT is due to inherent resistance to platin-based chemotherapy and the failure to clear all residual sites of disease after chemotherapy in the early treatment stages with omitted or improper post-chemotherapy surgery (PCS). The survival of patients with advanced GCT has improved over the past three decades which is attributed, in part, to improved risk stratification, delivery of risk-appropriate chemotherapy, improvements in second-line chemotherapy, expanding the role of PCS, and reduced treatment-related mortality.[4,5]

At diagnosis, approximately 50% and 5% of nonseminoma (NSGCT) and seminoma patients, respectively have evidence of bulky retroperitoneal or distant metastases, respectively. A small subset of patients with very high levels of the serum tumor markers (STM) alpha-fetoprotein (AFP), human choriogonadotropin (HCG), and lactate dehydrogenase (LDH), non-pulmonary visceral metastases, and mediastinal extragondal NSGCT have a poor prognosis which influences the choice of chemotherapy regimen and number of cycles. Other poor prognostic categories include those with malignant GCT at residual sites of disease after chemotherapy (particularly if an incomplete surgical resection is performed), patients with an incomplete response to first-line chemotherapy, and those who relapse after an initial complete response. The management of these patients has evolved considerably since the development of cisplatin-vinblastine-bleomycin (PVB), the initial cisplatin-based regimen. In this article, we will review the current management approaches for poor-prognosis patients at diagnosis, following chemotherapy, and at the time of early (< two years) and late (> two years) relapse.

### Poor-Prognosis GCT at Diagnosis

The prognostic classification of GCT patients at diagnosis is based on the International Germ Cell Cancer Collaborative Group (IGCCCG) criteria.[4] An international, retrospective pool of 5,202 patients with advanced NSGCT treated between 1975 and 1990 with platin-containing chemotherapy regimens was analyzed for prognostic factors for recurrence and survival. AFP, HCG, and LDH levels at the initiation of chemotherapy, the presence of non-pulmonary visceral metastasis, and primary mediastinal NSGCT were significant and independent prognostic factors for progression and survival.[4] In 660 patients with advanced seminoma, only the presence of non-pulmonary visceral metastasis was associated with survival.[4] Based on this analysis, the IGCCCG risk classification was developed [Table 1]. Approximately 28% and 16% of advanced NSGCT patients are classified as intermediate-and poor-risk by IGCCCG criteria and the five-year progression-free and overall survival rates for these patients is 75% and 80%, and 41% and 48%, respectively. Van Dijk *et al*. recently published a meta-analysis of 10 studies of 1775 NSGCT patients treated after 1989 and reported a substantially improved five-year survival of 83% and 71% for intermediate-and poor-risk patients.[5] There is no poor-risk category for advanced seminoma and approximately 10% are classified as intermediate-risk and the five-year survival for these patients is 72%.[4]

**Table 1:** International germ cell cancer collaborative group risk classification for advanced GCT.[4]

	NSGCT	Seminoma
Good-Risk	Testis/retroperitoneal primary	Any primary site
	and	and
	No non-pulmonary visceral metastasis	No non-pulmonary visceral metastasis
	and	and
	Good markers – all of	Normal AFP, any HCG, any LDH
	AFP < 1000 ng/mL	
	HCG < 5000 iu/L	
	LDH < 1.5× upper limit of normal	
Intermediate-Risk	Testis/retroperitoneal primary	Any primary site
	and	and
	No non-pulmonary visceral metastasis	Non-pulmonary visceral metastasis
	and	and
	Intermediate markers–any of	Normal AFP, any HCG, any LDH
	AFP 1000-10,000 ng/mL	
	HCG 5000-50,000 iu/L	
	LDH 1.5-10× upper limit of normal	
Poor-Risk	Mediastinal primary	No patients classified as poor-risk
	or	
	Non-pulmonary visceral metastasis	
	or	
	Poor markers – any of	
	AFP > 10,000 ng/mL	
	HCG > 50,000 iu/L	
	LDH > 10× upper limit of normal	

Since 1987, the standard approach for advanced GCT patients with intermediate-and poor-risk features has been BEPx4 chemotherapy after it was shown to have similar survival to PVBx4 but less neuromuscular toxicity.[6] Etoposide (VP-16)-ifosfamide-cisplatin (VIPx4) has been compared to BEPx4 in two randomized trials. The US trial failed to demonstrate a significant benefit of VIPx4 over BEPx4; the five-year survival (57% vs. 62%) was not significantly different but VIPx4 was associated with more serious hematological and genitourinary toxicity.[7,8] With 84 patients enrolled in the European trial, there were two GCT deaths in the BEPx4 arm and one in the VIPx4 arm, and overall survival at five years exceeded 80 percent.[9] Thus, BEPx4 has remained the standard regimen for intermediate-and poor-risk GCT.

High-dose chemotherapy (HDCT) using carboplatin-etoposide ± cyclophosphamide (CEC) with autologous stem cell support (also termed stem-cell rescue) has been investigated as an alternative to BEPx4 in patients with poor-prognosis GCT.[10–14] HDCT is based on the rationale that increasing dosage may overcome platin resistance. Carboplatin is used in HDCT regimens because of dose-limiting nephrotoxicity and neuropathy with cisplatin. A randomized trial of BEPx4 vs. BEPx2 plus two cycles of high-dose CEC in 219 patients with intermediate-(21%) and poor-risk GCT (79%) showed no significant difference in the one-year durable complete response rate (48% vs.52%, *P*=0.5) or overall survival.[15] The five-year survival for patients in both arms was 71% but toxicity was more severe for patients receiving HDCT. A smaller randomized trial also failed to demonstrate an improved survival with HDCT compared to standard-dose regimens as first-line therapy for patients with poor-prognosis metastatic GCT.[16] BEPx4 remains the standard first-line regimen in patients with intermediate-and poor-risk disease given the lack of superiority with ifosfamide-based standard-dose or high-dose regimens.

### Poor-Prognosis GCT after First-Line Chemotherapy

After first-line chemotherapy, approximately 5-15% of patients have disease progression or persistent marker elevation and these patients are managed with second-line chemotherapy (discussed later).[17–20] There is clear consensus that patients with marker normalization but with residual masses > 1 cm should undergo PCS.[21–24] The histology of resected specimens will demonstrate necrosis, teratoma, and viable malignancy (with or without teratoma) in 40%, 45%, and 15% of cases respectively.[18,19,25–34]

Compared to patients with necrosis or teratoma in PCS specimens, the presence of viable malignancy is associated with a poor outcome with reported five-year survival rates of 45-77% in patients who undergo a complete resection.[26–28,31–33,35–38] The role of postoperative chemotherapy in this setting is controversial. Fox *et al.*, reported that 14 of 27 patients (70%) undergoing PCS for viable malignancy were free of recurrence with adjuvant chemotherapy versus 0 of 7 patients who were observed.[36] In an international pooled analysis of 238 patients with viable malignancy in PCS specimens, Fizazi *et al*., identified pre-chemotherapy IGCCCG intermediate-and poor-risk disease, incomplete resection, and greater than 10% viable malignancy in PCS specimens as important prognostic factors.[35] Patients with 0, 1, and 2-3 risk factors had a five-year overall survival of 100%, 83%, and 51%, respectively. Overall, a significant improvement in five-year relapse-free survival was observed with postoperative chemotherapy (73% vs. 64%, *P*<0.001), but no difference in five-year overall survival (74% vs. 70%, *P*=0.7). In a subset analysis, patients with one risk factor had an improved five-year survival with postoperative chemotherapy (88% vs. 56%, *P*=0.02) but those with 0 (100% survival, with or without chemotherapy) and two to three risk factors (55% vs. 60%) did not. In a confirmatory study, this prognostic index was validated for relapse-free and overall survival and no significant difference in these endpoints was observed among the patients who did and did not receive postoperative chemotherapy.[38] A complete resection of residual masses is the most critical determinant of outcome for patients with viable malignancy in PCS specimens. Immediate postoperative chemotherapy or surveillance may be reasonable options depending on the completeness of resection, IGCCCG risk group, and percent of viable cells.

Approximately, 6-8% of PCS specimens will contain evidence of non-germ cell tumor malignancy, arising from malignant transformation of teratoma.[33,39,40] As with viable malignant GCT, the outcome of patients with malignant transformation is related to the completeness of surgical resection as they are generally resistant to GCT-specific chemotherapy regimens. With complete resection, approximately 50-66% of patients will survive, whereas the prognosis of those who have an incomplete resection is dismal.[39–43] Chemotherapy specific to the transformed histology (e.g. sarcoma-specific regimen) has been investigated in two small series in select patients with measurable disease limited to one histology. Partial responses were observed in a total of 11 of 24 patients, six of whom are alive with PCS.[44,45]

### Early Post-Chemotherapy GCT Relapse

Men who relapse after previously receiving first-line chemotherapy are treated with second-line chemotherapy. The majority of relapses will occur within two years of completing initial treatment and these are classified as early relapse.[7,9,15,46,47] Patients who fail to achieve a complete response to first-line therapy or who relapse within six months of achieving a complete response (termed incomplete responders) have a particularly poor prognosis.[48] In an international pooled analysis of 1984 patients from 38 centers with relapse after first-line chemotherapy, incomplete response to induction chemotherapy, primary mediastinal NSGCT, non-pulmonary visceral metastasis, and elevated serum tumor markers were associated with increased risk of progression with second-line chemotherapy. Overall, the three-year progression-free and overall survival was 38% and 51%, respectively.[14]

Conventional-dose regimens that have been studied in the second-and third-line settings include VIPx4, vinblastine-etoposide-cisplatin (VeIPx4; in men who had received prior etoposide from BEP regimens), and paclitaxel-ifosfamide-cisplatin (TIPx4). Studies of VIPx4 and VeIPx4 reported long-term remission rates of 23-35% and overall survival rates of 32-53%.[49–51] With TIPx4, relapse-free survival has been reported in 36-47% of patients.[52–54] TIPx4, VIPx4, and VeIPx4 have never been compared in a randomized trial and all are considered standard second-line regimens.

HDCT has also been investigated as second-line therapy in patients with GCT relapse, although its role in this setting is controversial. Indiana University has the largest, single-institution experience involving 184 patients with progression after first-(73%) or second-line chemotherapy (27%), 94% of whom received two or more courses of HDCT.[12] Over a median follow-up of four years, 63% of patients were continuously disease-free, including 70% and 45% of patients who received HDCT as second-and third-line therapy, respectively. An international matched-pair analysis comparing 74 patients treated at a single institution who received two to three cycles of VIP followed by one cycle of HDCT using carboplatin-etoposide-ifosfamide to 119 patients treated at multiple centers throughout Europe who received standard-dose, second-line chemotherapy using a variety of regimens, reported a 10% improvement in event-free and overall survival with HDCT.[55]

HDCT was compared to standard-dose, second-line chemotherapy in a randomized controlled trial enrolling 280 patients from 43 institutions. Patients in the standard-dose arm received VIPx4 or VeIPx4 and the HDCT arm received VIP/VeIPx3 followed by one cycle of high-dose CEC.[50] Over a median follow-up of 45 months, there were no significant differences in complete and partial response rates (56% in both arms) or the three-year event-free (35% vs. 42%, *P*=0.16) and overall survival (53% in both arms).

There are several potential explanations for the lack of benefit of HDCT in the randomized trial despite the favorable results reported in the two non-randomized studies. First, the results from single-arm trials may be subject to selection bias from differences in case-mix. In addition, the results achieved at high-volume institutions with unique experience with HDCT may not be reproducible at other institutions. Alternatively, the treatment strategy employed in the randomized trial may have been suboptimal in that three cycles of standard-dose chemotherapy and only one cycle of HDCT were given. In the randomized trial, only 73% of patients assigned HDCT were able to receive it and toxic deaths on the HDCT arm were twice as common as the standard-dose arm (7% vs. 3%). In the Indiana University series, 94% of patients were able to receive two cycles of HDCT and the treatment-related death rate was 2.7%. While HDCT as second-line therapy can cure a significant number of patients, the failure to demonstrate an improvement in survival compared to standard-dose regimens in three randomized trials (two as first-line therapy and one as second-line therapy) suggests it should not be considered a standard approach. Currently, HDCT should only be offered at specialized centers.

Patients with serologic complete response to second-line chemotherapy with residual masses should undergo post-salvage chemotherapy surgical resection (PSCS). A complete resection of residual masses is feasible in only 56-72% of patients (compared to 85% or more after first-line therapy).[18,28,32,36,56] Viable malignancy, teratoma, and necrosis are found in 53%, 21%, and 26% of PSCS specimens, respectively. The reported five-year overall survival is 44-61%.[28,32,36,37] Patients with viable malignancy in PSCS specimens have a particularly poor prognosis and their survival is not improved with the use of postoperative chemotherapy.

Patients with progressive disease despite second-or third-line chemotherapy have a dismal prognosis. However, a highly select group of patients with rising STMs who are deemed to have resectable disease limited to a single site (usually the retroperitoneum) may be candidates for salvage surgery, commonly referred to as “desperation surgery”. Although published studies are limited to small, single-institution case series, 47-60% will have normalization of serum tumor markers postoperatively and long-term survival is reported in 33-57% of patients after desperation surgery +/-postoperative chemotherapy.[57–61]

### Late Post-Chemotherapy GCT Relapse

Late relapse after chemotherapy is defined as that occurring more than two years after treatment. Roughly, 3% of advanced GCT patients experience a late relapse.[62,63] The histology of late relapse is viable malignancy in 54-88%, teratoma in 12-28%, and malignant transformation in 10-20%.[47,64–67] Given that the majority of late relapses occur in the retroperitoneum (50-72%), failure to control the retroperitoneum in the initial treatment phase appears to be the greatest risk factor.[62,64–68] Until recently, late relapse has been associated with a worse prognosis than early relapses, though contemporary data suggests these patients may have a similar probability of cure. In general, late relapse is resistant to chemotherapy and the outcome is related to the ability to render patients disease-free by complete surgical resection.[62,65–69]

The importance of surgery is related to the fact that teratoma and malignant transformation are inherently chemo-insensitive and viable malignancy is usually present in the setting of prior chemotherapy (thereby, platin-resistant). Of 32 patients with late relapse at Indiana University who received chemotherapy, only six (19%) achieved a complete response and five of these are in complete remission (three of whom were chemotherapy-naïve). Post-chemotherapy surgery successfully rendered 18 (69%) of the remaining 26 patients free of disease, 12 (46%) of whom remained in complete remission. Thus, 72% of patients treated with chemotherapy (+/-surgery) were rendered disease-free and 53% are in complete remission. Of the 49 patients treated initially with surgery, 45 (92%) were rendered free of disease overall (22[45%] by surgery alone), 29 (59%) are in complete remission. Overall, 69 (85%) patients achieved a disease-free state and 58% are disease-free over a median follow-up of 25 months.[65] In the Memorial Sloan-Kettering experience, the five-year cancer-specific survival was 60% and patients who had a complete surgical resection at the time of late relapse (60%) had a significantly improved survival compared to those without complete resection (40%) (79% vs. 36%, *P*<0.001).[67] The presence of symptoms and multifocal disease at late relapse were associated with inferior survival. In a German study of 72 NSGCT patients with late relapse (71% of whom had received prior chemotherapy), 35 (49%) were in complete remission at last follow-up, most of whom were treated with a combination of chemotherapy and surgery.[68] The most favorable chemotherapy results for late relapse are with the TIP regimen. A study from Memorial Sloan-Kettering reported that seven of 14 patients achieved a durable complete response to TIP plus surgical resection.[52] An aggressive surgical approach with complete resection of all sites of disease is the most important component to the cure of patients with late relapse. Chemotherapy may be considered as the initial management strategy if STMs are elevated or if an initial surgical complete resection is not feasible.

### Re-operative Retroperitoneal Lymph Node Dissection

Patients who have an incomplete resection of residual viable malignancy after first-line chemotherapy have a dismal prognosis. Likewise, patients who relapse in the retroperitoneum after PC-RPLND are similarly disadvantaged in terms of their ability to be salvaged by second-line chemotherapy and/or surgery. In the Indiana University experience, the relapse rate after redo-RPLND was 52% (compared to 21% after initial PC-RPLND) and the disease-free survival was 55%.[37] In the Memorial Sloan-Kettering experience, the five-year cancer-specific survival after redo-RPLND after initial PC-RPLND was 56%.[70]

## SUMMARY

Currently, the outcome of patients with intermediate-and poor-risk GCT at diagnosis is optimized by the use of risk-appropriate chemotherapy and post-chemotherapy surgical resection of residual masses at all anatomic sites of disease. Currently, there is no role for HDCT in the first-line setting. Patients who progress on first-line chemotherapy or who relapse after an initial complete response also have a poor prognosis. In the setting of early relapse, the standard approach at most centers is conventional-dose, ifosfamide-based regimens (TIPx4 or VeIPx4) and post-chemotherapy resection of residual masses. The treatment of patients with late relapse is complete surgical resection whenever feasible. Salvage chemotherapy for late relapse may be used prior to surgery in patients where a complete resection is not feasible.

A critical component to the cure of patients with advanced GCT is the complete resection of all residual disease elements after chemotherapy. The poor outcome of patients with early and late relapsed GCT and re-operative RPLND demonstrates the lethal consequences that may arise from the failure to eradicate all residual disease elements after initial chemotherapy by omitting PCS or failing to perform a complete resection. It would appear that observation after first-line chemotherapy is acceptable only for the 25% of patients who achieve a complete response, yet rates of PCS in published series typically range from 26-51%.[18–20,46,71,72] In several series, between 16-41% of patients with residual masses failed to undergo PCS.[18,20,46,71] Incomplete resection may result from approaches that involve resection of the residual mass only, the use of unilateral modified templates (particularly those that omit the para-aortic region for right-sided tumors and the interaortocaval region for left-sided tumors), and/or the lack of surgeon experience or resolve.[73] The importance of PCS was highlighted in a randomized trial of BEPx3 vs. EPx4 in 257 men with good-risk metastatic NSGCT.[46] As part of this trial, PCS was not dictated by protocol and only 52% underwent PCS, which frequently involved resection of the residual mass only. Overall, 14 of 20 (70%) relapsing patients and seven of 14 (50%) of those who died from GCT either did not undergo PCS or relapsed in the retroperitoneum after an inadequate RPLND. This study and others suggest that a substantial proportion of deaths from GCT may be prevented by the appropriate integration of chemotherapy and surgery.[71]
